# Serological indication of chronic inflammatory demyelinating polyneuropathy as an extrahepatic manifestation of hepatitis E virus infection

**DOI:** 10.1038/s41598-024-70104-3

**Published:** 2024-08-20

**Authors:** S. Pischke, A. Kjasimov, T. Skripuletz, C. Casar, J. Bannasch, M. Mader, S. Huber, F. Konen, A. Wolski, T. Horvatits, S. Gingele, S. Peine, J. Hiller, T. Seeliger, G. Thayssen, M. Lütgehetmann, J. Schulze zur Wiesch, A. Golsari, M. Gelderblom

**Affiliations:** 1 I. Department of Medicine, University Medical Center Hamburg-Eppendorf, Martinistrasse 52, 20251 Hamburg, Germany; 2 German Center for Infection Research (DZIF), Hamburg-Lübeck-Borstel-Riems Partner Site, Hamburg, Germany; 3 Department of Neurology, Hannover Medical School, Hannover, Germany; 4 Institute for Microbiology and Hygiene, University Medical Centre Hamburg-Eppendorf, Hamburg, Germany; 5 Institute of Transfusion Medicine, University Medical Center Hamburg-Eppendorf, Hamburg, Germany; 6 Department of Neurology, University Medical Center Hamburg-Eppendorf, Hamburg, Germany

**Keywords:** HEV, Hepatitis E, CIDP, Myasthenia gravis, Anti-HEV-IgG, Gastroenterology, Neurology

## Abstract

Guillain–Barré syndrome and neuralgic amyotrophy have been associated with hepatitis E virus (HEV) genotype 3 infections, while myasthenia gravis (MG) has been associated with HEV genotype 4 infections. However, whether chronic inflammatory demyelinating polyneuropathy (CIDP) is associated with HEV infections has not been conclusively clarified yet. 102 CIDP patients, 102 age- and sex-matched blood donors, 61 peripheral neuropathy patients (non-CIDP patients), and 26 MG patients were tested for HEV and anti-HEV IgM and IgG. Sixty-five of the 102 (64%) CIDP patients tested positive for anti-HEV IgG and one (1%) for anti-HEV IgM. No other patient tested positive for ati-HEV IgM. In the subgroup of CIDP patients with initial diagnosis (without previous IVIG treatment), 30/54 (56%) tested positive for anti-HEV IgG. Anti-HEV rates were significantly lower in blood donors (28%), non-CIDP peripheral neuropathy patients (20%), and MG patients (12%). No subject tested positive for HEV viremia. CSF tested negative for in 61 CIDP patients (54 patients with primary diagnosis). The development of CIDP but not non-CIDP polyneuropathy may be triggered by HEV exposure in an HEV genotype 3 endemic region. The increased anti-HEV seroprevalence in CIDP patients is not a consequence of IVIG therapy.

## Introduction

Hepatitis E virus (HEV) infections can cause liver inflammation and hepatitis and are also linked to various neurological disorders and diseases affecting other organs^1^. HEV has also been shown to replicate in vitro in neuronal tissue^2^, and HEV has been repeatedly detected in the cerebrospinal fluid (CSF) of infected individuals with neurological symptoms^3–6^.

HEV affects both central and peripheral nervous system processes. It has been associated with Guillain-Barré syndrome (GBS) and neuralgic amyotrophy (NA)^7–10^. Furthermore, detectable HEV in the CSF can persist despite its clearance in blood and feces^11,12^. All of these studies were conducted in Europe, an area where the consumption of pork endemically transmits the HEV genotype 3^1^. Thus, the associations between various neurological diseases and HEV genotype 3 infections are now established^1^.

In contrast, a potential association between HEV infections and myasthenia gravis (MG) is still debated. Myasthenia gravis is an autoimmune disease characterized by muscle weakness and fatigue, seems to be mainly B-cell mediated, and is associated with various specific antibodies^13^. In 2014, Belbezier et al.^14^ reported the case of an immunocompetent woman with acute HEV infection and concurrent MG. In 2018, Wang et al.^15^ reported a remarkably high rate of HEV viraemic and anti-HEV-IgM-positive patients in a cohort of 188 patients with MG onset from Beijing. Five percent of these patients (n = 10) tested positive for anti-HEV-IgM, and four of these patients were HEV viremic. HEV Genotyping revealed the common Chinese genotype 4 in all of them. However, this finding has not yet been investigated in European MG patients who are at risk of exposure to HEV GT3 rather than GT4.

Chronic inflammatory demyelinating polyneuropathy (CIDP) is a rare neurological disorder of the peripheral nervous system^16^. CIDP is an immune-mediated neuropathy defined by clinical progression for more than two months and electrodiagnostic evidence of peripheral nerve demyelination^17^. The cause of CIDP is considered to be an autoimmune disorder. In contrast to GBS and NA, an association with HEV has not yet been proven for CIDP. CIDP is a rare disease affecting approximately 5 in 100,000 people^16^. It commonly occurs in the 6th and 7th decades of life, with males being more commonly affected than females^16^. CIDP patients suffer from a range of clinical symptoms^16^.

Frequently, CIDP patients develop proximal and distal weakness in their limbs for weeks to months. Difficulties in fine motor skills as well as sensory disturbances are typical. The course of CIDP often rapidly progresses within a few months and can be either continuous or relapsing. A recent study from southern Germany investigated the anti-HEV IgM and IgG seroprevalence in a broad and less well-defined cohort of 99 patients with various neurological diseases^18^. In a small subgroup of 28 patients with acute inflammatory demyelinating polyneuropathy (AIDP) or chronic inflammatory demyelinating polyneuropathy (CIDP), 25% tested positive or borderline for anti-HEV IgG. However, the study's authors did not specify how many patients had AIDP versus CIDP, leaving the anti-HEV IgG seroprevalence in CIDP patients unclear.

Furthermore, a recent study from Italy showed that patients with CIDP (n = 82) had a significantly higher seroprevalence (39%) than the general population (45/269, 17%). The authors concluded that the passive transfer of anti-HEV antibodies through intravenous immunoglobulins (IVIGs) is the most likely explanation for this phenomenon^19^.

This study aims to clarify the potential association between HEV exposure and various immunologically modulated neurological disorders of the peripheral nervous system, particularly CIDP, in Europe, where HEV genotype 3 is endemic. Particularly the role of anti-HEV IgG positivity in the context of previous IVIG transfusions should be investigated.

## Materials and methods

**Figure 1:**
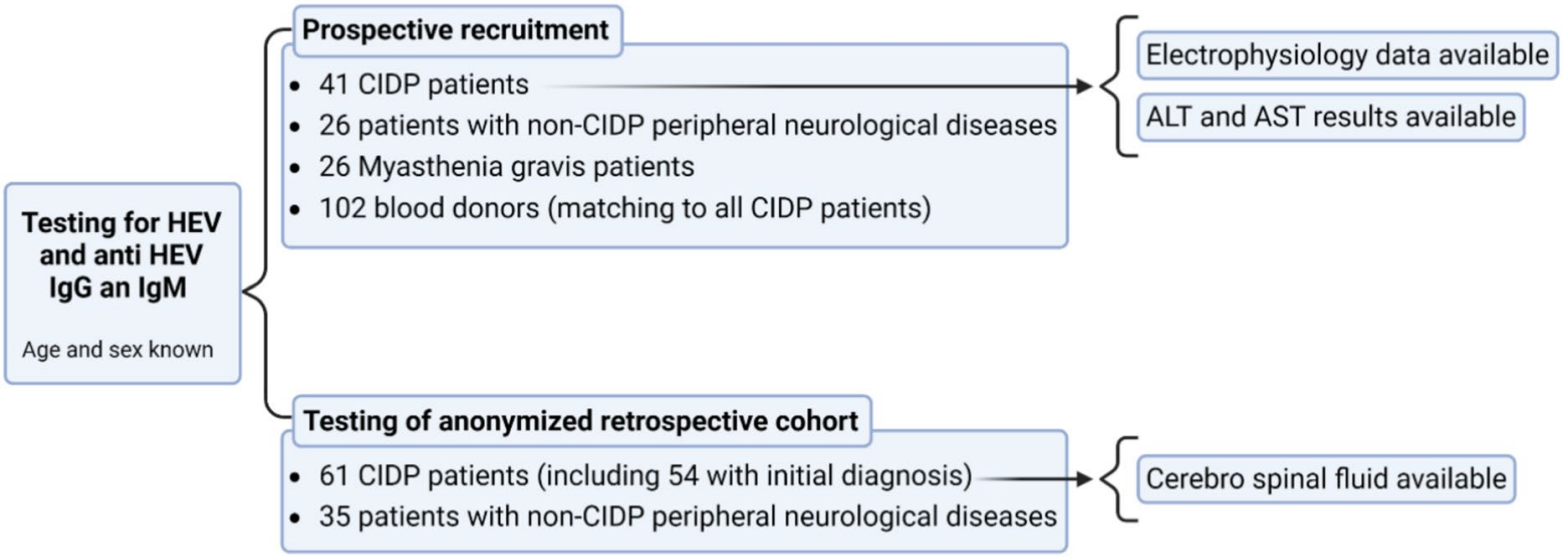
Study design and patient recruitment: 195 prospectively and 96 retrospectively studied individuals.

This study is prospective with retrospective components. The study design and patient recruitment of the prospective part are illustrated in Fig. 1, where 195 individuals were prospectively studied, including 41 CIDP patients from Hamburg, Germany. Furthermore, 91 individuals from Hannover, Germany, including 61 CIDP patients, were retrospectively studied. Hamburg and Hannover are located in northern Germany, are only 160 km apart, and share sociocultural and eating patterns. All patients came from the region of these two cities.

Patients and controls were prospectively recruited from the Hamburg area during the first nine months of 2021. For this cohort, all adult patients of the outpatient clinic for peripheral nervous diseases at the University Medical Center Hamburg-Eppendorf were unselectively invited to participate between May and September 2021. All these patients suffered from chronic peripheral nervous system diseases/polyneuropathies. 67 individuals (41 CIDP patients and 26 non-CIDP patients with peripheral neuropathy) provided written informed consent and were included in the study. Testing for anti-HEV IgG and IgM was conducted using Wantai ELISA tests (Wantai Bejing, China). To confirm anti-HEV IgG positivity, a blot (Mikrogen recomblot, Neuwied Germany) was used according to the manufacturer’s instructions, as described previously^20^. This commercial immunoblot not only confirmed the anti-HEV IgG serostatus, but also classified the antibody response according to HEV epitopes.

PCR was performed with a commercial assay from Altona Diagnostics (Hamburg, Germany).

Retrospective samples from Hannover, collected over the previous decade, were also analyzed after anonymization.

Additionally, plasma samples from 26 patients with myasthenia gravis (MG) from Hamburg and 102 age- and sex-matched blood donors from Hamburg were serologically tested for anti-HEV IgG and IgM, as well as HEV by PCR.

For organizational reasons, the 26 MG patients were not tested with the Altona diagnostic PCR but with the Roche Cobas TaqMan 6800 PCR (Roche, Hilden, Germany).

To validate the anti-HEV-IgG seroprevalence rate determined in the prospective cohort of 41 CIDP patients from the University Hospital Hamburg Eppendorf, a second cohort of 61 CIDP serum samples and 35 patients with non-CIDP neurological peripheral diseases from Hannover Medical School was tested for anti-HEV IgG and IgM, as well as HEV by PCR.

All CIDP patients (n = 102) were compared with an age- and sex-matched control group of 102 blood donors collected at the beginning of 2021. Due to the limited number of blood donors older than 70, exact matching was not possible, resulting in insignificant deviations.

Furthermore, all CIDP patients were tested for anti-CMV IgG (ELISA Viditest, MoBiTec, Goettingen, Germany). To further characterize the specificity of the anti-HEV IgG ELISA (Wantai) in the cohort of CIDP patients, the anti-CMV IgG seroprevalence in this group was compared with the anti-CMV seroprevalence of another cohort. This control group for anti-CMV IgG testing consisted of 553 patients with chronic liver or kidney diseases who were on waiting lists for organ transplantation, none of these patients previously received IVIG treatment.

To identify specific risk factors significantly associated with the previous HEV exposure 66 prospectively studied patients (40 CIDP, 26 non-CIDP) answered a questionnaire (Supplementary Table 1).

This study was approved by the local ethics committees of the Medical Council of Hamburg and the Hannover Medical School (WF-138/20, 9741_BO_S_2021). The study was conducted following the recommendations of the Declaration of Helsinki.

Statistical analysis was performed as follows: continuous variables with a nonnormal distribution are expressed as the median and interquartile range (IQR). Groups were compared using the Mann–Whitney U test. Categorical variables are expressed as numbers (%) and were compared with Fisher’s exact test. *P* values less than 0.05 were considered to indicate statistical significance. Statistical analyses were performed using SPSS, version 21.0 (IBM Corp., Armonk, NY, USA).

We utilized the R function glm (version 4.2.3, R Foundation for Statistical Computing, Vienna) for logistic regression with anti-HEV status as the response variable and patient group, age, and city of sample origin as independent variables.

## Results

**Figure 2:**
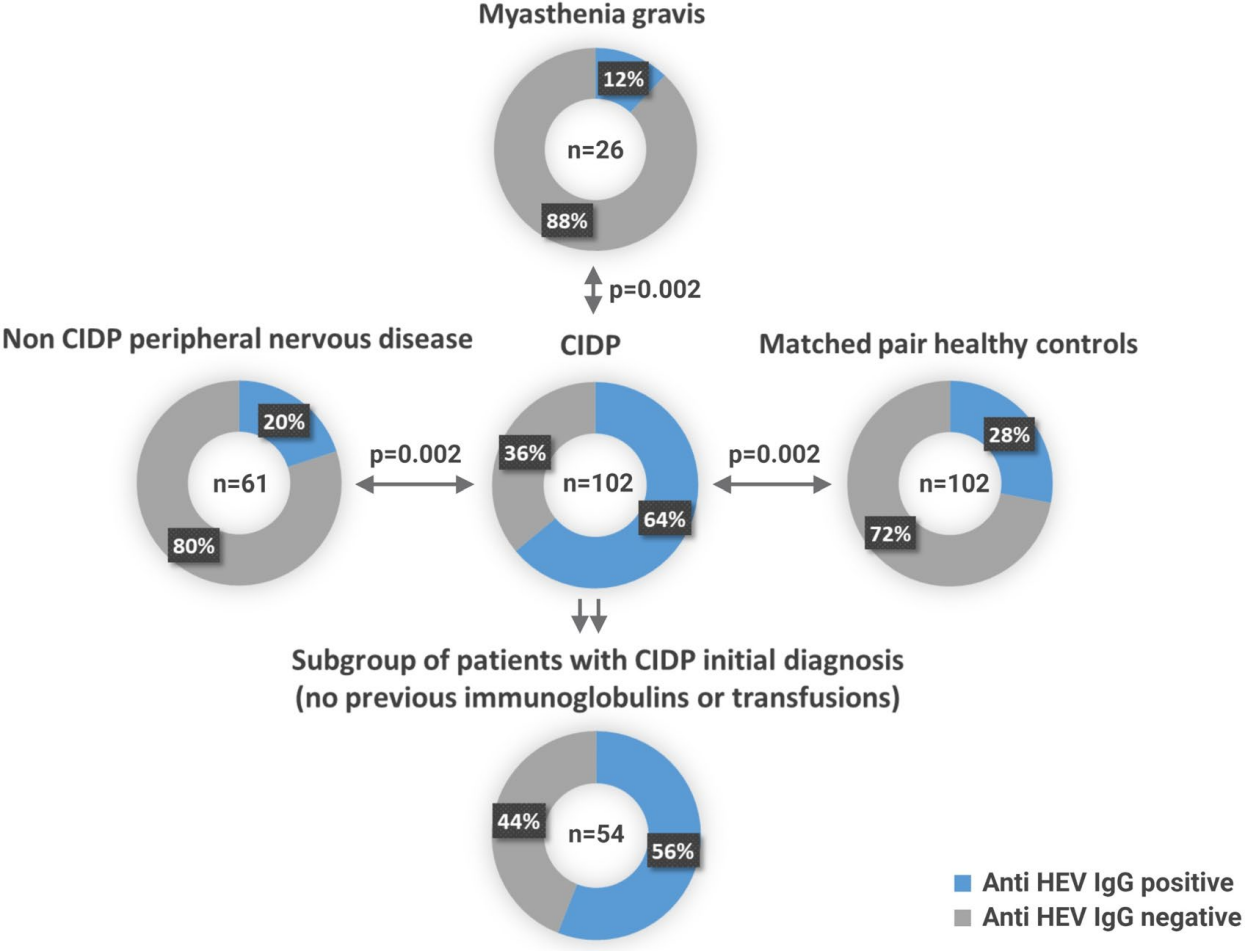
Anti-HEV seroprevalence in different cohorts.

As a main finding, significantly more patients with CIDP tested positive for anti-HEV-IgG (64%, 65/102) than did age- and sex-matched blood donors (28%, 29/102), patients with non-CIPD peripheral neuropathy (20%, 12/61) or MG patients (12%, 3/26) (*p* = 0.002 each; Fig. 2). None of these individuals tested positive for HEV RNA by PCR.

In detail, Sixty-one non-CIDP polyneuropathy patients had alcohol toxicity or diabetes-related polyneuropathy (n = 16), multifocal motoric neuropathy (n = 15), collagenosis-associated neuropathy (n = 7), paraneoplastic neuropathy (n = 4), or a single rare disease (n = 19). The seroprevalence of patients with non-CIDP polyneuropathy (20%) was not significantly different from that of MG patients (*p* = 0.55) or blood donors (*p* = 0.27). Furthermore, the seroprevalence among blood donors did not show a significant difference compared to that among MG patients (*p* = 0.13).

Motor and sensory nerve conduction studies (n = 41) from the prospective CIDP cohort, as well as epitope antibody profiling by blot, revealed no significant differences between anti-HEV-positive and anti-HEV-negative individuals (Tables 1 and 2). Unfortunately, these data were not available for the 61 retrospectively and anonymized studied CIDP patients (Fig. 1).

**Table 1:** Characteristics of prospectively studied CIDP patients (n = 41)*

	IgG positive (n = 31)	IgG negative (n = 10)	*p* value
Sex	23 male (75%)	6 male (60%)	0.5
Age, years	66	65	1.0
IgG total, g/l	7,69	6,53	0.2
ALT, U/ml	27	24	0.7
GM1 IgG, IgM	3	1	1.0
GM2	0	0	1.0
GM3	0	0	1.0
GQ1b IgG	0	2	1.0
GD1b IgM	0	0	1.0

*For data protection reasons, these data were not available for the retrospective cohort from Hannover.

**Table 2:** Nerve conduction velocity in 41 prospectively studied anti-HEV IgG-positive and -negative CIDP patients*

	Anti HEV IgG positive (n = 31)	Anti HEV IgG negative (n = 10)	*p* value
Motor studies			
Distal latency tibial (37/41)**, mean (Std. dev), ms	10.1 (20.7)	6.2 (1.6)	0.3
Distal latency peroneus (27/41)**, mean (Std. dev), ms	6.9 (3.9)	7.7 (4.8)	0.9
Distal latency ulnar 41/41)**, mean (Std. dev), ms	3.8 (1.7)	4.8 (3.0)	0.2
CMAP Amplitude tibial (37/41)**, mean (Std. dev), mV	2.7 (2.6)	1.9 (2.3)	0.6
CMAP Amplitude peroneus (26/41)**, mean (Std. dev), mV	1.8 (1.5)	2.1 (1.8)	0.8
CMAP Amplitude ulnar (41/41)**, mean (Std. dev), mV	5.5 (2.7)	5.6 (2.4)	0.9
Minimal F latency tibial (17/41)**, mean (Std. dev), ms	62.9 (13.5)	64.2 (6.2)	0.8
Minimal F latency peroneus (5/41)**, mean (Std. dev), ms	61.1 (10.1)	45.7 (40.0)	1.0
Minimal F latency ulnar (30/41) **, mean (Std. dev), ms	34.1 (4.7)	33.3 (6.9)	0.9
Sensory studies			
Conduction velocity sural (17/41)**, mean (Std. dev) m/s	42.0 (5.3)	38.9 (6.6)	0.5
Conduction velocity ulnar (22/41)**, mean (Std. dev), m/s	46.8 (7.4)	37.8 (10.5)	0.1
Amplitude sural (17/41)**, mean (Std. dev), µv	8.8.7 (7.1)	4.5 (2.4)	0.4
Amplitude ulnar (23/42)**, mean (Std. dev), µv	8.3 (5.6)	7.2 (4.8)	0.7

*For data protection reasons, these data were not available for the retrospective cohort from Hannover.

**Due to measurement difficulties and the retrospective assessment of these values, not all parameters were available for all subjects.

While total IgG levels were within the normal range in all patients with peripheral nerve diseases, total IgG levels were significantly greater in 86 anti-HEV-IgG-positive patients with peripheral nerve diseases (CIDP and non-CIDP) than in 76 negative patients with peripheral nerve diseases (CIDP and non-CIDP). In contrast, neither the total IgG level nor the CIDP-typical antibody pattern significantly differed in the prospectively studied group of 41 CIDP patients in the present study (Table 1).

The CIDP cohort consisted of 41 prospectively enrolled patients from Hamburg and 61 retrospectively studied patients from Hannover. The group of 61 retrospective patients (75% male, aged 32–81 years, mean age of 61 years) with stored and frozen serum samples included 54 patients with an initial diagnosis of CIDP. Among the patients who had never received IVIG, 56% (30/54) tested positive for anti-HEV IgG (Fig. 2). This rate (56%) represents a significantly increased seroprevalence compared to that in the blood donor control group (28%, *p* = 0.004), the non-CIDP cohort (20%, *p* = 0.002) or the MG patients (12%, *p* = 0.002).

Using logistic regression, we found a significantly lower chance of being anti-HEV positive in the healthy control group (OR = 0.105, 95% CI [0.041, 0.254], *p* < 0.001) and in the control group of non-CIDP polyneuropathy patients (OR = 0.094, 95% CI [0.033, 0.242], *p* < 0.001) than in the CIDP group. Age (OR = 1.01, 95% CI [0.989, 1.036], *p* > 0.05) and the city from which the virus originated (OR = 2.2, 95% CI [0.932, 5.49], *p* > 0.05) had no statistically significant effect on anti-HEV status.

Neither age nor time since initial diagnosis differed significantly between anti-HEV IgG-positive and -negative CIDP patients (supplementary Figs. 1 and 2).

All available serum and CSF samples (Fig. 1) tested PCR negative. All but one patient tested anti-HEV IgM negative. This anti-HEV IgM-positive patient was a 73-year-old woman with an initial diagnosis of CIDP. She had not previously received IVIG or other transfusions. However, 9 months earlier, the patient had a neurological disorder associated with isolated radial nerve damage, and the diagnosis of CIDP could not be made at that time. Fortunately, a serum sample from this episode 9 months before the initial diagnosis of CIDP was still available, and at that time, the patient tested negative for common HEV by PCR, as well as for anti-HEV IgM and anti-HEV IgG. Unfortunately, there was not enough material left to test the patient's blood sample from this time point for Rat-HEV.

Neither 9 months prior nor at the time of initial diagnosis did the patient have elevated AST or ALT values. No further patients, including those with non-CIDP polyneuropathy and MG tested positive by anti-HEV IgM test nor PCR.

To rule out the possibility that the anti-HEV IgG seroprevalence was due to nonspecific positivity from IVIG administration, we conducted an additional serology test for anti-CMV IgG. In the group of CIDP patients previously treated with IVIG, 98% were anti-CMV IgG positive (46/47). One patient did not have enough material for this test. In contrast, in the group of patients who were initially diagnosed with CIDP, without previous IVIG treatment, significantly fewer (50%) were anti-CMV IgG positive (27/54, *p* = 0.002). In a control group of 553 chronically ill liver or kidney patients (patients on the transplant waiting list, without previous exposure to IVIG), a comparable rate of 58% (323/553) were anti-CMV IgG positive (*p* = 0.3).

We validated the relevant positive anti-HEV IgG tests (Wantai test) in the Hannover CIDP subgroup with an initial diagnosis (no previous IVIG exposure) by testing available serum samples from 31 CIDP patients using a blot (Recomblot, Mikrogen, Neuried, Germany). In 30 of these patients (97%), anti-HEV positivity was confirmed. In contrast, anti-HEV IgG positivity was confirmed by the blot in only two out of four (50%) non-CIDP patients from the Hannover cohort (97% vs. 50%, *p* = 0.03, chi-square test).

Eighteen of the 30 confirmed anti-HEV IgG-positive (Wantai) CIDP patients showed one positive line (all reactive against O2CGT3), 10 tested positive for two lines (all reactive against O2CGT3 and O2cGT1), one patient tested positive for three lines (reactive against O2CGT3, O2CGT1, and O3GT1), and the last patient had four positive lines (reactive against O2CGT3, O2CGT1, O3GT1, and O3GT3) (Table 1).

To gain initial insights into whether anti-HEV IgG-positive and anti-HEV IgG-negative patients differ in terms of clinical severity or symptoms of neurological damage, neurophysiological measurements were prospectively studied in anti-HEV IgG-positive and anti-HEV IgG-negative CIDP patients (n = 41). However, no significant differences were found in this pilot study (Table 2).

We interviewed 66 patients with peripheral neuropathies to identify risk factors for contact with HEV and thus anti-HEV IgG seropositivity. The cohort included 40 patients with CIDP (61%) and 26 with non-CIDP (39%), with 38 testing positive for anti-HEV IgG (58%) and 28 testing negative (42%). The interviews covered diet, occupation, hobbies, sexual orientation, number of transfusions, and number of pets (Supplementary Table 1). No chi-square *p*-value for any of these parameters reached statistical significance. Specifically, pork consumption did not significantly differ between anti-HEV IgG-positive and anti-HEV IgG-negative individuals (*p* = 0.651).

## Discussion

Several studies have demonstrated an association between HEV infection and neurological conditions like NA or GBS, which are considered causative parainfectious autoimmune phenomena^1^. Our study clearly shows that patients with CIDP, another rare neurological inflammatory disease of the peripheral nervous system, have a significantly increased anti-HEV IgG seroprevalence (65%).

A recently published Italian study on 82 CIDP patients also found a significantly increased anti-HEV seroprevalence^19^, similar to our findings. However, unlike our study, the Italian researchers could not exclude IVIG products and transfused anti-HEV antibodies as potential explanations for this observation. Consequently, they interpreted this as the likely cause. CIDP patients generally receive treatment involving IVIG, plasmapheresis, and immunosuppression in various combinations, which have been shown to influence anti-HEV IgG seroprevalence^21–23^.

A detailed examination of a subgroup of patients with an initial CIDP diagnosis (n = 54) was performed in the current study to exclude the influence of these factors. This analysis revealed a significant increase in anti-HEV IgG positivity (56%) in this cohort, which had never received IVIG. In nearly 100% of these patients, IgG positivity was confirmed by a blot test. Therefore, prior exposure to HEV, rather than IVIG-mediated anti-HEV IgG antibodies, is indeed the cause of the heightened anti-HEV seropositivity in CIDP patients.

Although total IgG levels (Invitrogen) were within the normal range in the CIDP cohort, anti-CMV IgG was notably elevated, suggesting the likelihood of nonspecific false-positive serological results in CIDP patients previously treated with IVIG. Specifically, 98% of the 47 patients previously treated with IVIG tested positive for anti-CMV IgG, while only 50% (27/54) of the untreated cohort tested positive for anti-CMV. This seroprevalence (50%) was slightly lower than the 58% anti-CMV IgG seroprevalence observed in controls with chronic kidney or liver disease (323/553). Therefore, nonspecific serological findings are not a concern in untreated CIDP patients.

A perplexing observation in our study was that total IgG serum levels were higher in CIDP patients who tested positive for anti-HEV compared to those who tested negative, despite all IgG levels being within the normal range. This finding remains inconclusive. It is speculative whether a single previous anti-HEV IgG positive IVIG infusion influenced this phenomenon or if the immune systems of patients with prior HEV exposure were slightly more stimulated and tended to produce more antibodies. Future studies investigating the role of anti-HEV avidity and the HEV-specific T-cell response in anti-HEV positive versus negative CIDP patients will provide more insights.

Currently, it is unknown how many of the anti-HEV IgG-positive CIDP cases can be associated with prior HEV exposure, as false-positive results cannot be entirely ruled out. However, the rate of anti-HEV IgG positivity in the total cohort of CIDP patients (65%) and in the subgroup of untreated CIDP patients, who had never been exposed to IVIG, does not differ significantly but is substantially elevated compared to our control groups and the previously reported seroprevalence of 17% in the adult population in Germany^24,25^. Therefore, we conclude that prior IVIG therapy does not explain the increased anti-HEV IgG seroprevalence in CIDP patients. However, it remains undetermined to what extent, if any, IVIG has led to positive anti-HEV IgG test results in individual patients without prior HEV exposure.

Furthermore, not all antibodies against other viruses are generally increased in CIDP patients with previous IVIG therapy, as the anti-CMV seroprevalence was not increased. Notably, a previous study in patients with common variable immunodeficiency (CVID) receiving IVIG showed that anti-HEV OD values increased after IVIG infusion but did not reach the positive cut-off^26^. Additionally, during the COVID-19 pandemic, although anti-SARS-CoV-2 IgG was found in several IVIG preparations, the anti-SARS-CoV-2 IgG serum levels in recipients of these IVIGs were not significantly altered after infusion^26^. Based on these considerations, it is questionable whether conventional IVIG transfusions can significantly influence the seroprevalence of a single pathogen, including HEV, in a substantial number of patient groups.

Additionally, to ensure that the increased anti-HEV seroprevalence among CIDP patients is not limited to Hamburg, we analyzed a second cohort of CIDP patients from Hannover (Fig. 1). By using two separate cohorts from centers in northern Germany, located 160 km apart, we confirmed that CIDP patients have a significantly higher anti-HEV IgG seroprevalence.

Future studies should investigate larger, multicenter cohorts and longitudinally test both serology and HEV-specific T-cell responses from the onset of CIDP to gain further insights into the dynamics of the disease.

Of note, in the current study, one patient with CIDP tested positive for anti-HEV IgM, suggesting a potential link between HEV exposure and CIDP development. While this single case should not be overinterpreted, it might illustrate a plausible sequence of events: initial HEV exposure leading to mild neurological symptoms, seroconversion, and subsequent CIDP emergence. This would shed light on the relationship between HEV infections and neurological diseases. Initially, it was hypothesized that neurological disorders might stem from viral replication in neuronal cells. However, CIDP, typically considered autoimmune, challenges this view. Traditionally, autoimmunity was thought to target myelin sheaths, but our findings suggest HEVs may play a direct role, possibly initiating misdirected immune responses. This study is also pertinent to understanding myasthenia gravis (MG). While a previous Chinese study linked HEV genotype 4 to MG, our European study found no such association with HEV genotype 3, prevalent in our region. Nonetheless, our cohort of 26 MG patients may not definitively exclude an association, warranting larger studies for validation.

In summary, our study clearly demonstrates an association between HEV exposure and CIDP, a rare immune-mediated neurological disease. We confirm previous findings and show that elevated anti-HEV IgG levels in CIDP patients are not linked to IVIG treatment. However, our study has limitations that future research should address: our cohort of 102 CIDP patients, while substantial for this condition, calls for larger multicenter studies for validation. Additionally, future investigations should explore the immunological findings in more detail (e.g. analysis of the HEV-specific T-cell and B-cell responses, Antibody fine specificity, affinities, and cross-reactivity, or the T and B cell repertoires) since the findings of the current study are solely based on serological findings. Further subgroup analyses did not identify specific clinical or serological features unique to CIDP patients with prior HEV exposure, suggesting a need for larger and more diverse cohorts, including appropriate controls. Moreover, extending research to other neurological diseases, including those treated with IVIG, will help clarify whether previous HEV exposure is specific to CIDP or common across various conditions.

### Supplementary Information

Supplementary Information.

## Data Availability

Due to privacy and ethical concerns, neither the data nor the source of the data can be made available for confidential patient details. However, the corresponding author (S. Pischke) will happily answer any questions you may have.
