# Supplementary material for: Serological indication of chronic inflammatory demyelinating polyneuropathy as an extrahepatic manifestation of hepatitis E virus infection
Source: Sci Rep. 2024 Aug 20;14:19244. doi: 10.1038/s41598-024-70104-3 (PMC11336122; doi:10.1038/s41598-024-70104-3)
Supplement: Supplementary file 1 — Supplementary Information. [file 41598_2024_70104_MOESM1_ESM.pdf]

Supplementary Table 1: Distribution of various possible HEV exposure factors (based on a questionnaire)

	CIDP, anti-HEV IgG positive (n=30)	CIDP, anti-HEV IgG negative (n=10)	non-CIDP, anti-HEV IgG positive (n=8)	non-CIDP, anti-HEV IgG negative (n=18)
Male	21 (70%)	6 (60%)	8 (100%)	13 (72%)
Born in Germany	28 (93%)	6 (60%)	6 (75%)	12 (67%)
Traveling outside of Europe within the last 5 years	9 (30%)	5 (50%)	2 (25%)	8 (44%)
Previous transfusions	9 (30%)	3 (30%)	1 (13%)	4 (22%)
Pork consumption	28 (93%)	8 (80%)	6 (75%)	15 (83%)
Raw pork consumption	12 (40%)	2 (20%)	1 (13%)	4 (22%)
Game consumption	20 (67%)	7 (70%)	6 (75%)	13 (72%)
Mussels consumption	17 (57%)	9 (90%)	6 (75%)	9 (50%)
Raw beef consumption	12 (40%)	3 (30%)	5 (63%)	8 (44%)
Horse consumption	5 (17%)	1 (10%)	3 (38%)	2 (11%)
Sheep consumption	17 (57%)	7 (70%)	6 (75%)	8 (44%)
Raw fish consumption	8 (27%)	6 (60%)	6 (75%)	6 (33%)
Having a pet	8 (27%)	5 (50%)	1 (13%)	5 (28%)
Having kids	22 (73%)	8 (80%)	7 (88%)	10 (56%)
Working with pigs	3 (10%)	1 (10%)	0 (0%)	4 (22%)
Working in healthcare	4 (13%)	1 (10%)	2 (25%)	4 (22%)
Hobby: riding	0 (0%)	0 (0%)	1 (13%)	4 (22%)
Hobby: fishing	2 (7%)	1 (10%)	1(13%)	6 (33%)

There was no statistically significant difference according to the chi-square test. Sixty-six patients in the prospective CIDP and non-CIDP recruitment cohorts at Hamburg answered this questionnaire.

Supplemental figures

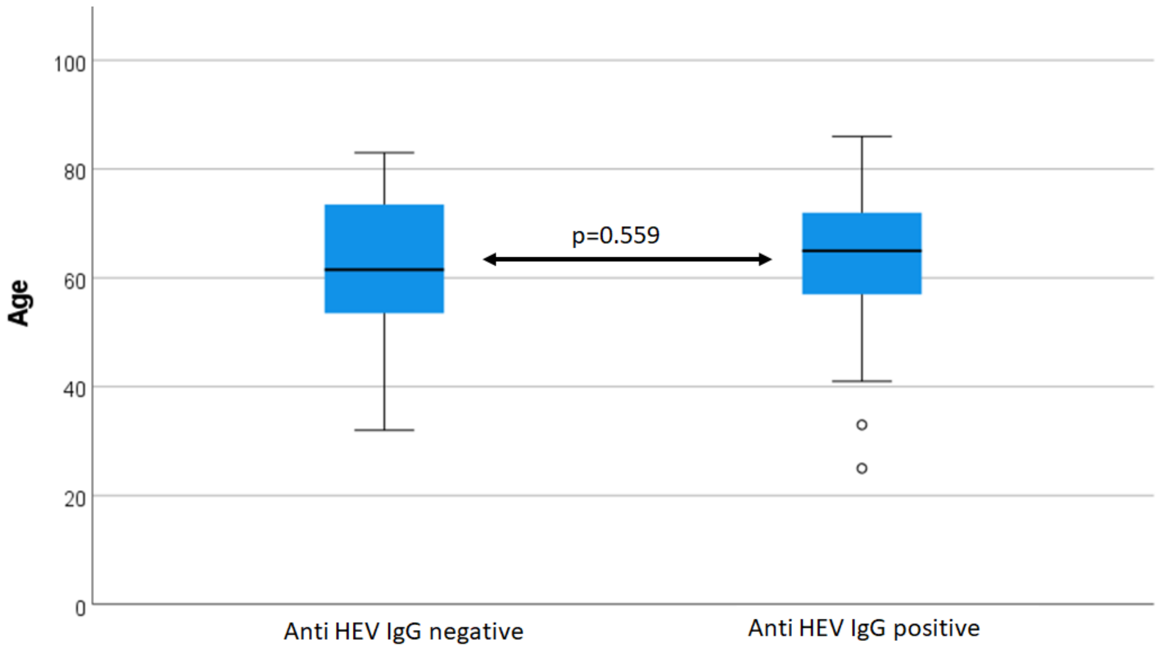

Supplementary figure 1: Age did not significantly differ between anti-HEV-IgG-positive CIDP patients and anti-HEV-IgG-negative CIDP patients (prospective cohort and retrospective cohort)

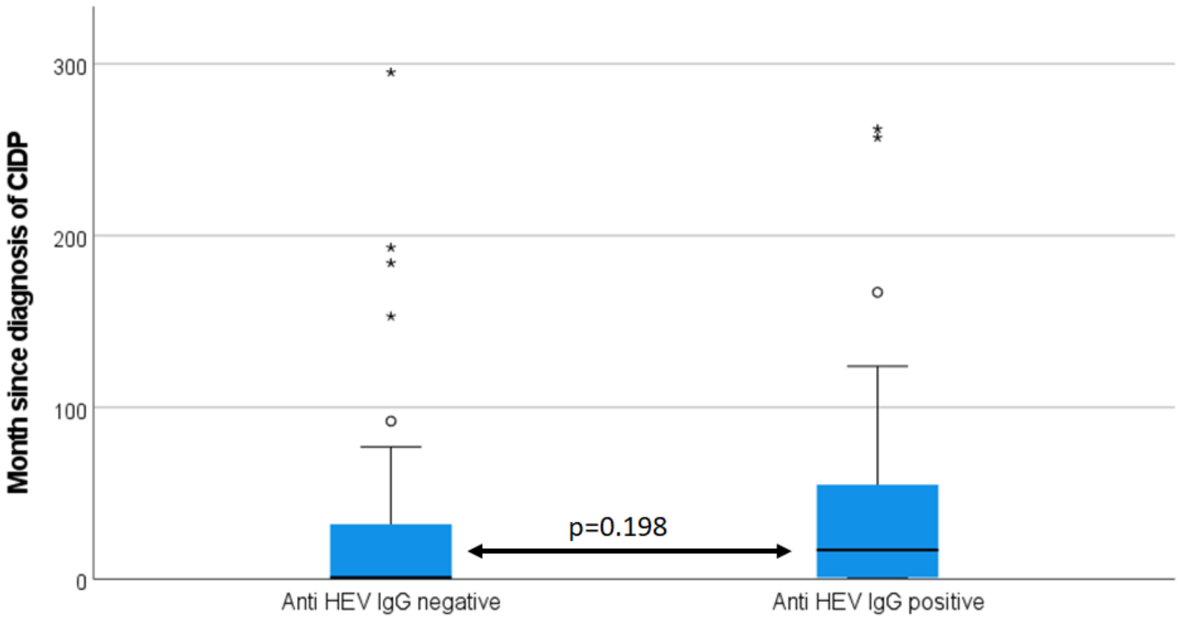

Supplementary figure 2: Time since diagnosis did not significantly differ between anti-HEV-IgG-positive and anti-HEV-IgG-negative CIDP patients (prospective and retrospective cohorts taken together).
